# Unrestricted consumption under a deterministic wealth and an Ornstein–Uhlenbeck process as a discount rate

**DOI:** 10.1080/15326349.2017.1392867

**Published:** 2018-01-09

**Authors:** Julia Eisenberg

**Affiliations:** ^a^ Institute of Mathematics and Scientific Computing, University of Graz, Graz, Austria; ^b^ Institute for Statistics and Mathematical Methods in Economics, TU Wien, Vienna, Austria

**Keywords:** Consumption, Hamilton–Jacobi–Bellman equation, optimal control, Ornstein–Uhlenbeck process, short rate, Primary: 93B05, Secondary: 49L20

## Abstract

We consider an individual or household endowed with an initial capital and an income, modeled as a linear function of time. Assuming that the discount rate evolves as an Ornstein–Uhlenbeck process, we target to find an unrestricted consumption strategy such that the value of the expected discounted consumption is maximized. Differently than in the case with restricted consumption rates, we can determine the optimal strategy and the value function.

## 1. Introduction

A pioneer of political economy Adam Smith said “Consumption is the sole end and purpose of all production; [...].” In fact, one of the fundamental questions in the decision theory is how an individual (or a household) should allocate her/his consumption over time and how much of an asset is it optimal to hold. The consumption behavior (to save or to consume) depends on various factors, but for the main part on the individual's wealth and on the asset price processes. There is a variety of models investigating the problem of optimal consumption/investment under different assumptions about the wealth and asset price processes, confer for example Refs.^[2,4,5]^ and references therein.

Basically, the considered individual has a choice between consuming her/his wealth or investing in an asset in order to maximize the expected consumption under a finite or infinite time horizon. Of course, the future cash flows should be transferred to the present through discounting. Usually, in order to simplify the calculations, the discount rate will be chosen as a deterministic constant, making the discount rate to the preference rate of the considered individual.

But what happens if the individual's consumption will be discounted by a stochastic process? The problem of stochastic discounting under a Brownian motion as a surplus process has been considered in Ref.^[3]^ There, it was possible to find explicit expressions for the value function and the optimal strategy if the discounting function was given by a geometric Brownian motion. In this special case, it turned out that the stochastic discounting did not change the optimal strategy significantly compared to the case with a constant preference rate. In the case, the discount rate was given by an Ornstein–Uhlenbeck process and restricted consumption rates it was shown that the value function was a viscosity solution to the problem; but neither the value function nor the optimal strategy had been found.

In the present paper, we assume that the wealth process of the considered individual before consumption is given by a linear function of time and the short rate process is given by an Ornstein–Uhlenbeck process. We target to find the optimal unrestricted consumption strategy, such that the expected discounted consumption is maximized. Of course, the assumption of a deterministic wealth process is not very realistic, but it allows to get a first idea of the influence of a stochastic interest rate on the consumption behavior. A detailed discussion of the advantages and disadvantages of a stochastic interest rate by consumption maximization problems would be very space-consuming and goes beyond the scope of this where we considered a similar problem with bounded consumption rates. However, there it was impossible to find an explicit expression for the value function or the optimal strategy due to some special properties of Ornstein–Uhlenbeck processes.

The remainder of the paper is organized as follows. In Section 2, we formulate the conjecture that the optimal strategy is of barrier type and state in the verification theorem that the value function is a classical solution to the Hamilton–Jacobi–Bellman (HJB) equation corresponding to the problem. In Section 3, we analyze the components of the value functions and prove the assumptions made in Section 2 to hold true. The results are illustrated by an example.

## 2. The model and the value function

Consider an individual or household with an income given by a deterministic linear function of time

$$X_{t}=x+\mu t\;,$$

μ ⩾ 0. Denote further by {*r_s_*} an Ornstein–Uhlenbeck process

$$r_{s}=re^{-as}+\tilde{b}\left(1-e^{-as}\right)+\tilde{\sigma }e^{-as}\int_{0}^{s}e^{au}\;dW_{u}\;,$$

where {*W_u_*} is a standard Brownian motion, $a,\tilde{\sigma }>0$, and let *U^r^_s_* = ∫^*s*^_0_ *r_u_* d*u* with *r*_0_ = *r*. Our target is to maximize the expected discounted consumption over all admissible strategies *C*, if the interest rate is given by {*r_t_*}. A strategy *C* is called admissible if *C* is non-decreasing, adapted to the filtration $\left\{F_{s}\right\}$ generated by {*r_s_*} and *X^C^_t_* = *X_t_* − *C_t_* ⩾ 0 for all *t* ⩾ 0.

Here, we assume that the long-term mean $\tilde{b}$ of the process {*r_s_*} fulfils: $\tilde{b}>\frac{{\tilde{\sigma }}^{2}}{2a^{2}}$ and define

$$b:=\tilde{b}-\frac{{\tilde{\sigma }}^{2}}{2a^{2}}\;\;\text{and}\;\;\sigma :=\frac{\tilde{\sigma }}{\sqrt{2a}}\;.$$

The return function corresponding to a strategy *C* and the value function are defined by

$$\begin{array}{ccc} V^{C}\left(r,x\right) & = & E\left[\int_{0}^{\infty }e^{-U_{s}^{r}}\;dC_{s}|X_{0}=x,r_{0}=r\right],\;\left(r,x\right)\in R\times R_{+}\;,\\ V(r,x) & = & \underset{C}{\sup}V^{C}\left(r,x\right),\;\left(r,x\right)\in R\times R_{+}\;. \end{array}$$

Note, that also lump sum payments are possible. The HJB equation corresponding to the problem is

(1) $$\max\left\{\mu V_{x}+a\left(\tilde{b}-r\right)V_{r}+\frac{{\tilde{\sigma }}^{2}}{2}V_{rr}-rV,1-V_{x}\right\}=0\;.$$

For the sake of convenience, we define an operator acting on sufficiently smooth functions

(2) $$L\left(f\right)\left(r,x\right):=\mu f_{x}\left(r,x\right)+a\left(\tilde{b}-r\right)f_{r}\left(r,x\right)+\frac{{\tilde{\sigma }}^{2}}{2}f_{rr}\left(r,x\right)-rf\left(r,x\right)\;.$$

Intuitively, it is clear that when starting with a negative initial discount rate, one should forego consumption, because the discounting factor $e^{-U_{s}^{r}}$ will increase at least until {*r_t_*} becomes positive. On the other hand, if *r*_0_ > 0 then due to $\tilde{b}>0$ it could happen that − *U^r^_s_* will remain negative and will keep decreasing in time. In this case, it would make sense to start consuming on the maximal rate immediately.

We conjecture that the optimal strategy would be of barrier type, i.e., it is optimal to consume if the short rate process exceeds some special value and to do nothing otherwise.

Let $r^{*}\in R$ be arbitrary but fixed and define

$$\tau :=\inf\left\{t\geq 0:\;r_{t}=r^{*},\;r_{0}=r<r^{*}\right\}\;\text{and}\;\varrho :=\inf\left\{t\geq 0:\;r_{t}=r^{*},\;r_{0}=r>r^{*}\right\}\;$$

and

$$\begin{array}{ccc} G(r,x) & := & E[\left(x+\mu \tau +G\left(\tilde{r},0\right)\right)e^{-U_{\tau }^{r}}]\;\text{for}\;r\leq r^{*},\\ F(r,x) & := & x+E\left[\mu \int_{0}^{\varrho }e^{-U_{s}^{r}}\;ds+G\left(r^{*},0\right)e^{-U_{\varrho }^{r}}\right]\;\text{for}\;r\geq r^{*}\;. \end{array}$$

Note that we distinguish between τ and ϱ just for the convenience of notation.

Obviously, $G_{x}\left(r,x\right)=E\left[e^{-U_{\tau }^{r}}\right]$ and *F_x_*(*r*, *x*) = 1. Then, it is clear *G*(r*, *x*) = *F*(r*, *x*) and *G_x_*(r*, *x*) = *F_x_*(r*, *x*).

Assumption. Assume now, r* is chosen in such a way that the functions *G* and *F* given above solve the HJB equation (1) on ( − ∞, r*] and on [r*, ∞) respectively and fulfil *G_r_*(r*, *x*) = *F_r_*(r*, *x*) for all $x\in R_{+}$. Then, we can formulate the following verification theorem.

Proposition 2.1. Let

$$\eta_{t}:=\sup\left\{s\in \left[0,t\right]:\;r_{s}\geq r^{*}\right\}\;\text{with}\;\eta_{t}=-\infty \;\text{if}\;\{s\in \left[0,t\right]:\;r_{s}\geq r^{*}\}=\emptyset \text{.}$$

The optimal strategy is to immediately consume any capital bigger than zero if *r* ⩾ r*, i.e., the optimal accumulated consumption up to time *t* is given by $C_{t}^{*}=1\;{\text{I}}_{\eta_{t}\geq 0}\left(x+\mu \eta_{t}\right)$. The value function *V*(*r*, *x*) is continuously differentiable with respect to *r* and to *x*, twice continuously differentiable with respect to *r* on $R\setminus \left\{r^{*}\right\}\times R_{+}$ and fulfils *V*(*r*, *x*) = *v*(*r*, *x*) with

$$v\left(r,x\right)=\left\{\begin{array}{cc} G(r,x) & :\left(r,x\right)\in \left(-\infty ,r^{*}\right]\times R_{+}\\ F(r,x) & :\left(r,x\right)\in \left(r^{*},\infty \right)\times R_{+} \end{array}\right..$$

Proof. For the convenience of reading, we postpone the proof to the end of the paper.

**Figure 1.**
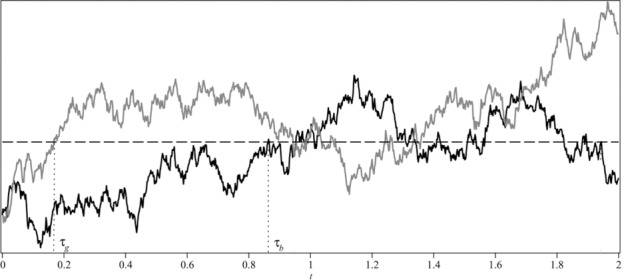
Realizations of *r_t_*.

Remark 2.1. At first glance, the formal representation of the optimal strategy may appear confusing. Assume *r*_0_ = *r* < r* (the case *r*_0_ ⩾ r* goes analogously). In Figure 1, one sees two realizations of the process {*r_t_*}: gray and black, up to time *t* = 2. The first hitting times of the optimal level r* are denoted by τ_*g*_ and τ_*b*_, respectively. Note that C*_t_ = 0 for *t* < τ_*g*_ in the case of the gray realization or for *t* < τ_*b*_ in the case of the black realization. In the periods where *r_t_* < r*, the accumulated optimal strategy remains constant. As soon as the process reaches r*, the saved capital is paid out at once. Thus, if η_*t*_ = *t* then we have paid out *x* + μ*t* up to time *t*. For instance, in the gray realization it holds η_2_ = 2 meaning C*_2_ = *x* + 2μ. However, in the black realization η_2_ = 1.9 implies C*_2_ = C*_1.9_ = *x* + 1.9μ.

## 3. Analysis of the value function

In the previous section, we assumed the existence of a barrier r* such that the corresponding return function

$$v\left(r,x\right)=\left\{\begin{array}{cc} G(r,x) & :\left(r,x\right)\in \left(-\infty ,r^{*}\right]\times R_{+}\\ F(r,x) & :\left(r,x\right)\in \left(r^{*},\infty \right)\times R_{+} \end{array}\right..$$

solves the HJB equation (1) and fulfils *G*(r*, *x*) = *F*(r*, *x*), *G_r_*(r*, *x*) = *F_r_*(r*, *x*) and *G_x_*(r*, *x*) = *F_x_*(r*, *x*).

In the present section, we prove that the assumptions made in the previous section hold true. The section is structured as follows: at first, we investigate the properties of *G* and *F* for a given but unknown r*, then letting the barrier r* be a variable, we derive a method for finding the optimal and unique optimal barrier. Thus, we start by considering

(3) $$\begin{array}{ccc} \psi_{1}\left(r\right) & := & E\left[e^{-U_{\tau }^{r}}\right],\;\;\;r\in \left(-\infty ,r^{*}\right],\\ \psi_{2}\left(r\right) & := & E\left[\tau e^{-U_{\tau }^{r}}\right],\;\;\;\;r\in \left(-\infty ,r^{*}\right],\\ \varphi_{1}\left(r\right) & := & E\left[e^{-U_{\varrho }^{r}}\right],\;\;r\in \left[r^{*},\infty \right),\\ \varphi_{2}\left(r\right) & := & E\left[\int_{0}^{\varrho }e^{-U_{s}^{r}}\;ds\right],\;\;\;r\in \left[r^{*},\infty \right)\;. \end{array}$$

Due to the properties of {*r_t_*}, the hitting times τ and ϱ are finite a.s. Note that $U_{t}^{r}=\frac{r-r_{t}}{a}+\tilde{b}t+\frac{\tilde{\sigma }}{a}W_{t}$. Thus, using the change of measure techniques, compare for instance Ref. [7, p. 216], with $\frac{\;dP}{\;dQ}=\exp\left(\frac{\tilde{\sigma }}{a}W_{\tau }+\frac{{\tilde{\sigma }}^{2}}{2a^{2}}\tau \right)$ one obtains

$$\begin{array}{ccc} \psi_{1}\left(r\right) & = & E\left[e^{-U_{\tau }^{r}}\right]=e^{-\frac{r-r^{*}}{a}}E\left[e^{-\tilde{b}\tau -\frac{\tilde{\sigma }}{a}W_{\tau }}\right]=e^{-\frac{r-r^{*}}{a}}E_{Q}\left[e^{-\tilde{b}\tau -\frac{{\tilde{\sigma }}^{2}}{2a^{2}}\tau }\right]\\ & = & e^{-\frac{r-r^{*}}{a}}E_{Q}\left[e^{-b\tau }\right]\;. \end{array}$$

Under the measure *Q*, the process {*r_t_*} has the long-term mean $b=\tilde{b}-\frac{{\tilde{\sigma }}^{2}}{2a^{2}}$. In order to calculate $E[e^{-U_{\tau }^{r}}]$, we have to consider the Laplace transform of τ. A parabolic cylinder function is defined as

$$\begin{array}{ccc} D_{-v}\left(y\right) & = & e^{-y^{2}/4}2^{-v/2}\sqrt{\pi }\\ & & \times \;\left\{\frac{1+\sum_{k=1}^{\infty }\prod_{j=0}^{k-1}\frac{\left(v+2j\right)y^{2k}}{(2k)!}}{\Gamma (\frac{v+1}{2})}-\frac{y\sqrt{2}(1+\sum_{k=1}^{\infty }\prod_{j=0}^{k-1}\frac{\left(v+2j+1\right)y^{2k}}{(2k+1)!})}{\Gamma (v/2)}\right\}, \end{array}$$

confer for example Borodin and Salminen [1, p. 639]. By ${\tilde{D}}_{v}\left(y\right)$ we denote in the following the parabolic cylinder function *D*_− *v*_(*y*) multiplied by $e^{y^{2}/4}$. In Ref. [1, pp. 542], one also finds the following formula

$$\begin{array}{c} E_{Q}\left[e^{-b\tau }\right]=\frac{e^{\frac{{\left(r-b\right)}^{2}}{4\sigma^{2}}}D_{-b/a}(-\frac{r-b}{\sigma })}{e^{\frac{{\left(r^{*}-b\right)}^{2}}{4\sigma^{2}}}D_{-b/a}(-\frac{r^{*}-b}{\sigma })}=\frac{{\tilde{D}}_{b/a}(-\frac{r-b}{\sigma })}{{\tilde{D}}_{b/a}(-\frac{r^{*}-b}{\sigma })}\;, \end{array}$$

implying

$$\begin{array}{c} \frac{\;d}{\;dr}E_{Q}\left[e^{-b\tau }\right]=\frac{b}{a\sigma }\frac{{\tilde{D}}_{b/a+1}(-\frac{r-b}{\sigma })}{{\tilde{D}}_{b/a}(-\frac{r^{*}-b}{\sigma })}=E_{Q}\left[e^{-(b+a)\tau }\right]\frac{b}{a\sigma }\frac{{\tilde{D}}_{b/a+1}(-\frac{r^{*}-b}{\sigma })}{{\tilde{D}}_{b/a}(-\frac{r^{*}-b}{\sigma })}\;, \end{array}$$

so that we can calculate $E[e^{-U_{\tau }^{r}}]$ explicitly. Nevertheless, an explicit representation will not help us to obtain the properties of the value function.

Remark 3.1. The functions defined in (3) fulfil the following boundary conditions.

- For *r* = r*, it holds τ = ϱ = 0, yielding ψ_1_(r*) = 1, ψ_2_(r*) = 0, ϕ_1_(r*) = 1, and ϕ_2_(r*) = 0.
- Let ε > |r*|, *r* < −ε, and τ^ε^ ≔ {*t* ⩾ 0: *r_t_* = −ε, *r*_0_ = *r*}. Then,
  $$\psi_{1}\left(r\right)=E\left[e^{-U_{\tau^{\varepsilon }}^{r}}\right]\psi_{1}\left(-\varepsilon \right)\geq E\left[e^{\tau^{\varepsilon }\varepsilon }|r_{0}=r\right]\psi_{1}\left(-\varepsilon \right)\;.$$
  Since τ^ε^ → ∞ as *r* → −∞, we obtain $\lim_{r\to -\infty }\psi_{1}\left(r\right)=\infty$. In the same way, one can show that $\lim_{r\to -\infty }\psi_{2}\left(r\right)=\infty$.
- Due to the representation
  $$\varphi_{1}\left(r\right)=e^{-\frac{r-r^{*}}{a}}E_{Q}\left[e^{-b\varrho }\right]\;,$$
  one obtains $\lim_{r\to \infty }\varphi_{1}\left(r\right)=\lim_{r\to \infty }\varphi_{2}\left(r\right)=0$.

Similar to Shreve et al.^[8]^, we formulate the following lemma.

Lemma 3.1. The functions ψ_1_ and ϕ_1_ solve differential equation

(4) $$\begin{array}{c} a\left(\tilde{b}-r\right)f^{'}\left(r\right)+\frac{{\tilde{\sigma }}^{2}}{2}f^{''}\left(r\right)-rf\left(r\right)=0\;, \end{array}$$

the function ϕ_2_ solves

(5) $$1+a\left(\tilde{b}-r\right)f^{'}\left(r\right)+\frac{{\tilde{\sigma }}^{2}}{2}f^{''}\left(r\right)-rf\left(r\right)=0\;,$$

and ψ_2_ solves

(6) $$\psi_{1}\left(r\right)+a\left(\tilde{b}-r\right)f^{'}\left(r\right)+\frac{{\tilde{\sigma }}^{2}}{2}f^{''}\left(r\right)-rf\left(r\right)=0\;.$$

Under the boundary conditions

$$\begin{array}{ccc} \psi_{1}\left(r^{*}\right) & = & 1,\;\lim_{r\to -\infty }\psi_{1}\left(r\right)=\infty ,\\ \psi_{2}\left(r^{*}\right) & = & 0,\;\lim_{r\to -\infty }\psi_{2}\left(r\right)=\infty ,\\ \varphi_{1}\left(r^{*}\right) & = & 1,\;\lim_{r\to \infty }\varphi_{1}\left(r\right)=0,\\ \varphi_{2}\left(r^{*}\right) & = & 0,\;\lim_{r\to \infty }\varphi_{2}\left(r\right)=0. \end{array}$$

Proof. We prove the statement for ψ_1_, the proof for ϕ_1_ follows with the same techniques.

- Let *f* be a solution to Equation
  $$\frac{{\tilde{\sigma }}^{2}}{2}f^{''}\left(r\right)+a\left(b-r\right)f^{'}\left(r\right)=bf\left(r\right)\;,$$
  on ( − ∞, r*] with boundary conditions *f*(r*) = 1 and $\lim_{r\to -\infty }f\left(r\right)=0$. Such a solution exists due to the Picard–Lindelöf theorem. In particular, using $\lim_{r\to -\infty }f\left(r\right)=0$ yields that *f*′(*r*) is bounded on ( − ∞, r*]. Then, by Ito's lemma (using that under the measure *Q*, the process {*r_t_*} has the long-term mean *b*), denoting by $\tilde{W}$ the new Brownian motion under *Q*:
  $$\begin{array}{ccc} e^{-b\tau \wedge t}f\left(r_{\tau \wedge t}\right) & = & f\left(r\right)+\int_{0}^{\tau \wedge t}e^{-bs}\left\{a\left(b-r_{s}\right)f^{'}\left(r_{s}\right)+\frac{{\tilde{\sigma }}^{2}}{2}f^{''}\left(r_{s}\right)-bf\left(r_{s}\right)\right\}\;ds\\ & & +\;\tilde{\sigma }\int_{0}^{\tau \wedge t}e^{-bs}f^{'}\left(r_{s}\right)\;d{\tilde{W}}_{s}\\ & = & f\left(r\right)+\tilde{\sigma }\int_{0}^{\tau \wedge t}e^{-bs}f^{'}\left(r_{s}\right)\;d{\tilde{W}}_{s}\;. \end{array}$$
  The integrand in the stochastic integral is bounded for *r_s_* ∈ ( − ∞, r*] so that the expectation of the stochastic integral equals zero, giving $f\left(r\right)=E_{Q}\left[e^{-b(\tau \wedge t)}f\left(r_{\tau \wedge t}\right)\right]$. The above differential equation implies that max {*f*(*r*): *r* ∈ ( − ∞, r*]} < ∞. Letting now *t* → ∞ and noting that by Lebesgue's dominated convergence theorem limit and integration can be interchanged, we obtain
  $$f\left(r\right)=E_{Q}\left[e^{-b\tau }\right]\;.$$
  It can be easily shown that the function $\psi_{1}\left(r\right)=e^{-\frac{r-r^{*}}{a}}E_{Q}\left[e^{-b\tau }\right]$ solves Equation (4) with the corresponding boundary conditions.
- Let now *f*(*r*) solve Equation (5) with the boundary conditions $\lim_{r\to \infty }f\left(r\right)=0$ and *f*(r*) = 0. Ito's formula yields
  $$\begin{array}{c} e^{-U_{\varrho \wedge t}^{r}}f\left(r_{\varrho \wedge t}\right)=f\left(r\right)-\int_{0}^{\varrho \wedge t}e^{-U_{s}^{r}}\;ds+\tilde{\sigma }\int_{0}^{\varrho \wedge t}e^{-U_{s}^{r}}f^{'}\left(r_{s}\right)\;dW_{s}, \end{array}$$
  giving $f\left(r\right)=E[e^{-U_{\varrho \wedge t}}f\left(r_{\varrho \wedge t}\right)+\int_{0}^{\varrho \wedge t}e^{-U_{s}^{r}}\;ds]$. Let now *t* → ∞. Again by Lebesgue's dominated convergence theorem, we obtain
  $$f\left(r\right)=E\left[\int_{0}^{\varrho }e^{-U_{s}^{r}}\;ds\right]\;.$$
- Let $h\left(r\right):=E_{Q}\left[e^{-b\tau }|r_{0}=r\right]$. Assume, *f* solves the equation
  $$h\left(r\right)+\frac{{\tilde{\sigma }}^{2}}{2}f^{''}\left(r\right)+a\left(b-r\right)f^{'}\left(r\right)=bf\left(r\right)$$
  with the boundary conditions $\lim_{r\to -\infty }f\left(r\right)=0$ and *f*(r*) = 0. Due to the differential equation and to $\lim_{r\to \infty }f\left(r\right)=0$, the functions *f* and *f*′ are bounded. By Ito's formula (under the measure *Q*)
  $$\begin{array}{c} e^{-b(\tau \wedge t)}f\left(r_{\tau \wedge t}\right)=f\left(r\right)-\int_{0}^{\tau \wedge t}e^{-bs}h\left(r_{s}\right)\;ds+\tilde{\sigma }\int_{0}^{\tau \wedge t}e^{-bs}f^{'}\left(r_{s}\right)\;d{\tilde{W}}_{s}. \end{array}$$
  The expectation of the stochastic integral is equal to zero. For the first integral on the right side of the above equation, one gets due to the Markov property of {*r_t_*}:
  $$\begin{array}{ccc} E_{Q}\left[\int_{0}^{\tau \wedge t}e^{-bs}h\left(r_{s}\right)\;ds\right] & = & \int_{0}^{\infty }E_{Q}\left[1\;{\text{I}}_{\left[s\leq \tau \wedge t\right]}e^{-bs}h\left(r_{s}\right)\right]\;ds\\ & = & \int_{0}^{\infty }E_{Q}\left[1\;{\text{I}}_{\left[s\leq \tau \wedge t\right]}E_{Q}[e^{-b\tau }|r_{s}]\right]\;ds\\ & = & \int_{0}^{\infty }E_{Q}\left[E_{Q}\left[1\;{\text{I}}_{\left[s\leq \tau \wedge t\right]}e^{-b\tau }|F_{s}\right]\right]\;ds=E_{Q}\left[\left(\tau \wedge t\right)e^{-b\tau }\right]\;. \end{array}$$
  Letting *t* → ∞ and using Lebesgue's dominated convergence theorem yields the desired result. It is easy to show that the function $\psi_{2}\left(r\right)=e^{-\frac{r-r^{*}}{a}}E_{Q}\left[\tau e^{-b\tau }\right]$ solves Equation (6).

Remark 3.2. Lemma 3.1 implies that the functions *F* and *G* are twice continuously differentiable with respect to *r*, once continuously differentiable with respect to *x* on $\left(r^{*},\infty \right)\times R_{+}$ and on $\left(-\infty ,r^{*}\right)\times R_{+}$ respectively and fulfil there

$$\begin{array}{ccc} \mu F_{x}\left(r,x\right)+a\left(\tilde{b}-r\right)F_{r}\left(r,x\right)+\frac{{\tilde{\sigma }}^{2}}{2}F_{rr}\left(r,x\right)-rF\left(r,x\right) & = & -rx\;,\\ \mu G_{x}\left(r,x\right)+a\left(\tilde{b}-r\right)G_{r}\left(r,x\right)+\frac{{\tilde{\sigma }}^{2}}{2}G_{rr}\left(r,x\right)-rG\left(r,x\right) & = & 0\;. \end{array}$$

In particular, *F* solves the HJB equation on $\left[r^{*},\infty \right)\times R_{+}$ if r* ⩾ 0. The function *G* solves the HJB equation on ( − ∞, r*] if ψ_1_(*r*) < 1 for *r* < r* and ψ_1_(r*) = 1.

### 3.1. The function *G*

Note further that *e* ^− *b*τ^τ, *r* < r*, is finite, so that one has

ψ2(r)=E[e-Uτrτ]=e-r-r*aEQ[e-bττ]=e-r-r*aEQ1b∑n=1∞1n∑k=0nnk(-1)ke-bτ(k+1)=e-r-r*ab∑n=1∞1n∑k=0nnk(-1)kD˜b(k+1)/a-r-bσD˜b(k+1)/a(b-r*σ).

Thus, for the function *G*, we find

G(r,x)=(x+G(r*,0))e-r-r*aD˜b/a(-r-bσ)D˜b/a(b-r*σ)+μe-r-r*ab∑n=1∞1n∑k=0nnk(-1)kD˜b(k+1)/a(-r-bσ)D˜b(k+1)/a(b-r*σ).

In order to find an explicit expression for the function *F*, we have to find ϕ_1_ and ϕ_2_. For ϕ_1_, it holds due to [1, pp. 542]

$$\varphi_{1}\left(r\right)=E\left[e^{-U_{\varrho }^{r}}\right]=e^{-\frac{r-r^{*}}{a}}E_{Q}\left[e^{-b\varrho }\right]=e^{-\frac{r-r^{*}}{a}}\frac{{\tilde{D}}_{b/a}(\frac{r-b}{\sigma })}{{\tilde{D}}_{b/a}(\frac{r^{*}-b}{\sigma })}\;.$$

To find ϕ_2_, we have to solve differential equation (5) and determine the coefficients with the help of the corresponding boundary conditions $\lim_{r\to \infty }\varphi_{2}\left(r\right)=0=\varphi_{2}\left(r^{*}\right)$.

### 3.2. The optimal strategy

In order to obtain a continuously differentiable solution, we have to guarantee that the first derivatives of *F* and *G* coincide on $\left\{\left(r^{*},x\right),\;x\in R_{+}\right\}$. Obviously, it holds *G*(r*, *x*) = *F*(r*, *x*), *G_x_*(r*, *x*) = *F_x_*(r*, *x*). The derivative of *F* with respect to *r* does not depend on *x*. In order to have a continuously differentiable solution with respect to *r*, the derivative *G_r_*(r*, *x*) should not depend on *x*. Thus, we have three conditions, yielding a continuously differentiable with respect to *x* and to *r* function, solving the HJB equation on $R\setminus \left\{r^{*}\right\}\times R_{+}$:

- *G_rx_*(r*, *x*) = ψ′_1_(r*) = 0,
- $E[e^{-U_{\tau }^{r}}]>1$ for all *r* < r*,
- r* ⩾ 0.

It holds

$$\begin{array}{ccc} G_{rx}\left(r,x\right)=\psi_{1}^{'}\left(r\right) & = & \frac{\;d}{\;dr}e^{-\frac{r-r^{*}}{a}}E_{Q}\left[e^{-b\tau }\right]=\frac{\;d}{\;dr}e^{-\frac{r-r^{*}}{a}}\frac{{\tilde{D}}_{b/a}(\frac{b-r}{\sigma })}{{\tilde{D}}_{b/a}(\frac{b-r^{*}}{\sigma })}\\ & = & -\frac{1}{a}e^{-\frac{r-r^{*}}{a}}\frac{{\tilde{D}}_{b/a}(\frac{b-r}{\sigma })}{{\tilde{D}}_{b/a}(\frac{b-r^{*}}{\sigma })}+\frac{b}{a\sigma }e^{-\frac{r-r^{*}}{a}}\frac{{\tilde{D}}_{b/a+1}(\frac{b-r}{\sigma })}{{\tilde{D}}_{b/a}(\frac{b-r^{*}}{\sigma })}\\ & = & -\frac{1}{a}e^{-\frac{r-r^{*}}{a}}E_{Q}\left[e^{-b\tau }\right]+\frac{b}{a\sigma }e^{-\frac{r-r^{*}}{a}}\frac{{\tilde{D}}_{b/a+1}(\frac{b-r^{*}}{\sigma })}{{\tilde{D}}_{b/a}(\frac{b-r^{*}}{\sigma })}E_{Q}\left[e^{-(b+a)\tau }\right]\\ & = & e^{-\frac{r-r^{*}}{a}}E\left[e^{-b\tau }\left(-\frac{1}{a}+\frac{b}{a\sigma }\frac{{\tilde{D}}_{b/a+1}(\frac{b-r^{*}}{\sigma })}{{\tilde{D}}_{b/a}(\frac{b-r^{*}}{\sigma })}e^{-a\tau }\right)\right]\;. \end{array}$$

Thus, if $\frac{{\tilde{D}}_{b/a+1}\left(\frac{b-r^{*}}{\sigma }\right)}{{\tilde{D}}_{b/a}\left(\frac{b-r^{*}}{\sigma }\right)}=\frac{\sigma }{b}$ then ψ′_1_(r*) = 0 and ψ_1_(*r*) is strictly decreasing in *r*, which implies ψ_1_(*r*) > 1, for all *r* < r*.

In order to show the existence and uniqueness of r* with the properties described above, we have to consider the function

$$H\left(y\right):=\frac{{\tilde{D}}_{b/a+1}(\frac{b-y}{\sigma })}{{\tilde{D}}_{b/a}(\frac{b-y}{\sigma })}\;.$$

Since it is impossible, to determine the properties of the functions $\tilde{D}$ directly, we will derive the properties of *H* from the differential equation corresponding to $\tilde{D}$.

Lemma 3.2. The function $H:R\to R_{+}$ is strictly increasing, surjective and $H\left(0\right)<\frac{\sigma }{b}$.

Proof. Similar to Shreve et al.^[8]^, it has been shown in Lemma 3.1 that the function $h\left(r\right):=E\left[e^{-b\tau }\right]$ solves the following equation

(7) $$\frac{{\tilde{\sigma }}^{2}}{2}h^{''}\left(r\right)+a\left(b-r\right)h^{'}\left(r\right)=bh\left(r\right)$$

with boundary conditions *h*(r*) = 1 and $\lim_{r\to -\infty }h\left(r\right)=0$. Due to the properties of τ, the function *h* is strictly increasing. For the same reason, *h*′(*r*) is strictly increasing:

$$h^{'}\left(r\right)=\frac{b}{a\sigma }\frac{{\tilde{D}}_{b/a+1}(\frac{b-r}{\sigma })}{{\tilde{D}}_{b/a}(\frac{b-r^{*}}{\sigma })}=h^{'}\left(r^{*}\right)E\left[e^{-(b+a)\tau }\right]\;.$$

Since *b* > 0, the function *h*(*r*) does not have real zeros. Dividing (7) by *h*(*r*) yields

(8) $$\frac{{\tilde{\sigma }}^{2}}{2}\frac{h^{''}\left(r\right)}{h(r)}+a\left(b-r\right)\frac{h^{'}\left(r\right)}{h(r)}=b\;.$$

Note that $H\left(r\right)=\frac{\sigma a}{b}\frac{h^{'}\left(r\right)}{h(r)}$.

Letting *r* → −∞ on the left side of the above equation yields $\lim_{r\to -\infty }\frac{h^{'}\left(r\right)}{h(r)}=0$ because otherwise the left-hand side would become infinite. Thus, we can conclude

$$\lim_{r\to -\infty }H^{'}\left(r\right)=\frac{\sigma a}{b}\lim_{r\to -\infty }\left\{\frac{h^{''}\left(r\right)}{h(r)}-\frac{h^{'}{\left(r\right)}^{2}}{h{\left(r\right)}^{2}}\right\}\geq 0\;.$$

On the other hand, we can rewrite the above equation in terms of *H* and its derivatives

$$\frac{{\tilde{\sigma }}^{2}}{2}H^{'}\left(r\right)=b-a\left(b-r\right)H\left(r\right)-\frac{{\tilde{\sigma }}^{2}}{2}H{\left(r\right)}^{2}\;,$$

which means $\frac{{\tilde{\sigma }}^{2}}{2}H^{''}\left(r\right)=-a\left(b-r\right)H^{'}\left(r\right)-{\tilde{\sigma }}^{2}H\left(r\right)H^{'}\left(r\right)+aH\left(r\right)$. According to this, one has *H*′′(*r*) > 0 if *H*′(*r*) = 0, which implies *H*′(*r*) > 0 for r∈R due to $\lim_{r\to -\infty }H^{'}\left(r\right)\geq 0$.

- It holds $\lim_{r\to \infty }\frac{h^{'}\left(r\right)}{h(r)}=\infty$. Assume first $\lim_{r\to \infty }\frac{h^{'}\left(r\right)}{h(r)}=-A>-\infty$ for some $A\in R_{+}$, i.e., $\lim_{r\to \infty }\frac{h^{'}\left(r\right)}{h(r)}=0$, which contradicts *H*′(*r*) > 0. Assume now $\lim_{r\to \infty }\frac{h^{'}\left(r\right)}{h(r)}=B<\infty$, which gives $\lim_{r\to \infty }H^{'}\left(r\right)=0$. But Equation (8) yields $\lim_{r\to \infty }\frac{h^{''}\left(r\right)}{h(r)}=\infty$, giving $\lim_{r\to \infty }H^{'}\left(r\right)=\lim_{r\to \infty }\left\{\frac{h^{''}\left(r\right)}{h(r)}-\frac{h^{'}{\left(r\right)}^{2}}{h{\left(r\right)}^{2}}\right\}=\infty$.
  Thus, $\lim_{r\to -\infty }H\left(r\right)=0$, $\lim_{r\to -\infty }H\left(r\right)=\infty$. By the intermediate value theorem, we can conclude that *H*(*r*) attains every value in $R_{+}$.
  Inserting *r* = 0 into Equation (8) and multiplying (8) by $\frac{\sigma }{b^{2}}$, yields
  $$\begin{array}{ccc} \frac{\sigma }{b} & = & \frac{{\tilde{\sigma }}^{2}\sigma }{2b^{2}}\frac{h^{''}\left(0\right)}{h(0)}+\frac{\sigma a}{b}\frac{h^{'}\left(0\right)}{h(0)}=\frac{{\tilde{\sigma }}^{2}\sigma }{2b^{2}}\frac{h^{''}\left(0\right)}{h(0)}+H\left(0\right)\;. \end{array}$$
  Since $\frac{{\tilde{\sigma }}^{2}\sigma }{2b^{2}}\frac{h^{''}\left(0\right)}{h(0)}>0$, it holds $H\left(0\right)<\frac{\sigma }{b}$.

Due to the above lemma, there is a unique r* > 0 such that $H\left(r^{*}\right)=\frac{\sigma }{b}$, meaning that *G* solves the HJB equation on $\left(-\infty ,r^{*}\right]\times R_{+}$ and *F* solves the HJB equation on $\left[r^{*},\infty \right)\times R_{+}$.

Letting $G\left(r^{*},0\right)=\mu \frac{\psi_{2}^{'}\left(r^{*}\right)-\varphi_{2}^{'}\left(r^{*}\right)}{\varphi_{1}^{'}\left(r^{*}\right)}$ guarantees *G_r_*(r*, *x*) = *F_r_*(r*, *x*) for all $x\in R_{+}$. It remains to show that

Lemma 3.3. The constant

$$\Delta :=\mu \frac{\psi_{2}^{'}\left(r^{*}\right)-\varphi_{2}^{'}\left(r^{*}\right)}{\varphi_{1}^{'}\left(r^{*}\right)}$$

is positive and finite.

Proof. Using the same change of measure technique like in the beginning of Section 2, we obtain

$$\begin{array}{c} \varphi_{1}\left(r\right)=E\left[e^{-U_{\varrho }^{r}}\right]=e^{-\frac{r-r^{*}}{a}}E_{Q}\left[e^{-b\varrho }\right]\;. \end{array}$$

The stopping time ϱ is increasing in *r*, implying that ϕ_1_(*r*) is strictly decreasing in *r*, i.e., ϕ′_1_(r*) < 0.

As for the function ϕ_2_, it holds

$$\varphi_{2}\left(r\right)=E\left[\int_{0}^{\varrho }e^{-U_{s}^{r}}\;ds\right]=e^{-\frac{r}{a}}E\left[\int_{0}^{\varrho }e^{\frac{r}{a}e^{-as}-U_{s}^{0}}\;ds\right]\;,$$

meaning that $e^{\frac{r}{a}}\varphi_{2}\left(r\right)$ is strictly increasing in *r*. Thus, using that ϕ_2_(r*) = 0:

$$\varphi_{2}^{'}\left(r^{*}\right)=-\frac{1}{a}\varphi_{2}\left(r^{*}\right)+e^{-\frac{r}{a}}\frac{\;d}{\;dr}\left(e^{\frac{r}{a}}\varphi_{2}\left(r\right)\right){\left|{}_{r=r^{*}}=e^{-\frac{r}{a}}\frac{\;d}{\;dr}\left(e^{\frac{r}{a}}\varphi_{2}\left(r\right)\right)\right|}_{r=r^{*}}>0\;.$$

We can rewrite the function ψ_2_ like in the proof of Lemma 3.1

$$\begin{array}{c} \psi_{2}\left(r\right)=E\left[\int_{0}^{\tau }e^{-U_{s}^{r}}\psi_{1}\left(r_{s}\right)\;ds\right]\;. \end{array}$$

By the definition of r*, the function ψ_1_(*r*) is strictly decreasing in *r*, which means that ψ_1_(*r_s_*) is strictly decreasing in *r*. Since, τ and − *U^r^_s_* are strictly decreasing in *r*, we can conclude that ψ_2_(*r*) is strictly decreasing, which proves the claim.

Remark 3.3. Note that the optimal boundary r* does not depend on the drift μ. This roots in μ ⩾ 0: the “good interest rates” are good independent of the available income and non-negative income rate. The situation would change if one allows the income rate to become negative: the “good interest rates” can coincide with the periods of decreasing income. However, *G*(r*, 0) (discounted accumulated future consumption) naturally depends on μ.

Proof of Proposition 2.1. Let *C* be an arbitrary admissible strategy. Applying the fundamental theorem of calculus yields

(9) $$\begin{array}{c} v\left(r_{t},X_{t}^{C}\right)=v\left(r_{t},x\right)+\int_{0}^{t}v_{x}\left(r_{t},X_{s}^{C}\right)\;dX_{s}^{C}\;. \end{array}$$

In the following, we examine the two terms on the right side of the above equation. Ito's formula requires *v* to be twice continuously differentiable with respect to *r*, which is not fulfilled for *r* = r* and *x* > 0. Therefore, we use the extant second derivative Meyer–Ito formula [6, p. 221] where we just need *v* to have an absolutely continuous derivative with respect to *r* and *v_rr_* to be locally *L* ^1^. Since *F_r_*(r*, *x*) = *G_r_*(r*, *x*) = 1 for all $x\in R_{+}$, it is an easy exercise to verify that *v* satisfies all above requirements. Then,

(10) $$\begin{array}{c} v\left(r_{t},x\right)=v\left(r,x\right)+\int_{0}^{t}v_{r}\left(r_{s},x\right)\;dr_{s}+\frac{{\tilde{\sigma }}^{2}}{2}\int_{0}^{t}v_{rr}\left(r_{s},x\right)\;ds\;. \end{array}$$

Before, we consider *v_x_*(*r_t_*, *X^C^_s_*), note that *v_x_* does not depend on *x* and it holds either *v_x_* = 1 or *v_x_* = ψ_1_. In particular, one can interchange the derivation order, i.e., *v_rx_* = *v_xr_* and *v_rrx_* = *v_xrr_*. Like *v*, the function *v_x_* fulfils the conditions of the extant second derivative Meyer–Ito formula [6, p. 221]:

$$\begin{array}{ccc} v_{x}\left(r_{t},X_{s}^{C}\right) & = & v_{x}(r_{s},X_{s}^{C})+\int_{s}^{t}v_{xr}(r_{y},X_{s}^{C})\;dr_{y}+\frac{{\tilde{\sigma }}^{2}}{2}\int_{s}^{t}v_{xrr}(r_{y},X_{s}^{C})\;dy\\ & = & v_{x}\left(r_{s},x\right)+\int_{s}^{t}v_{rx}(r_{y},X_{s}^{C})\;dr_{y}+\frac{{\tilde{\sigma }}^{2}}{2}\int_{s}^{t}v_{rrx}(r_{y},X_{s}^{C})\;dy\;. \end{array}$$

Thus, integrating the above equality from 0 to *t* with respect to  d*X^C^_s_* and applying Fubini's theorem yields

(11) $$\begin{array}{ccc} & & \int_{0}^{t}v_{x}(r_{t},X_{s}^{C})\;dX_{s}^{C}\\ & & \;=\int_{0}^{t}\left\{v_{x}(r_{s},X_{s}^{C})+\int_{s}^{t}v_{rx}(r_{y},X_{s}^{C})\;dr_{y}+\frac{{\tilde{\sigma }}^{2}}{2}\int_{s}^{t}v_{rrx}(r_{y},X_{s}^{C})\;dy\right\}\;dX_{s}^{C}\\ & & \;=\int_{0}^{t}v_{x}(r_{s},X_{s}^{C})\;dX_{s}^{C}+\int_{0}^{t}\{v_{r}(r_{y},X_{y}^{C})-v_{r}\left(r_{y},x\right)\}\;dr_{y}\\ & & \;+\;\frac{{\tilde{\sigma }}^{2}}{2}\int_{0}^{t}\{v_{rr}(r_{y},X_{y}^{C})-v_{rr}\left(r_{y},x\right)\}\;dy\;. \end{array}$$

Thus, inserting (10) and (11) into (9) yields

$$\begin{array}{ccc} v\left(r_{t},X_{t}^{C}\right) & = & v\left(r,x\right)+\int_{0}^{t}v_{r}(r_{s},X_{s}^{C})\;dr_{s}+\frac{{\tilde{\sigma }}^{2}}{2}\int_{0}^{t}v_{rr}(r_{s},X_{s}^{C})\;ds+\int_{0}^{t}v_{x}(r_{s},X_{s}^{C})\;dX_{s}^{C}\\ & = & v\left(r,x\right)+\int_{0}^{t}\mu v_{x}(r_{s},X_{s}^{C})+a\left(\tilde{b}-r_{s}\right)v_{r}(r_{s},X_{s}^{C})+\frac{{\tilde{\sigma }}^{2}}{2}v_{rr}(r_{s},X_{s}^{C})\;ds\\ & & +\;\tilde{\sigma }\int_{0}^{t}v_{r}(r_{s},X_{s}^{C})\;dW_{s}-\int_{0}^{t}v_{x}(r_{s},X_{s}^{C})\;dC_{s}\;. \end{array}$$

Via the product rule, using *v_x_*(*r_s_*, *X^C^_s_*) ⩾ 1 and *L*(*v*)(*r_s_*, *X^C^_s_*) ⩽ 0, we obtain

$$\begin{array}{ccc} e^{-U_{t}^{r}}v\left(r_{t},X_{t}^{C}\right) & = & v\left(r,x\right)+\int_{0}^{t}e^{-U_{s}^{r}}L\left(v\right)(r_{s},X_{s}^{C})\;ds+\tilde{\sigma }\int_{0}^{t}v_{r}(r_{s},X_{s}^{C})\;dW_{s}\\ & & -\;\int_{0}^{t}e^{-U_{s}^{r}}v_{x}(r_{s},X_{s}^{C})\;dC_{s}\\ & \leq & v\left(r,x\right)+\tilde{\sigma }\int_{0}^{t}e^{-U_{s}^{r}}v_{r}(r_{s},X_{s}^{C})\;dW_{s}-\int_{0}^{t}e^{-U_{s}^{r}}\;dC_{s}\;, \end{array}$$

with *L* defined in (2). Note that for the strategy C* equality holds. Consider the stochastic integral above. Recap that $U_{s}^{r}=\frac{r-r_{s}}{a}+\tilde{b}s+\frac{\tilde{\sigma }}{a}W_{s}$. Further, due to the properties of the functions *G* and *F* the functions $e^{\frac{r-r^{*}}{a}}G_{r}$ and $e^{\frac{r-r^{*}}{a}}F_{r}$ are bounded. In particular

$$\begin{array}{c} e^{-U_{s}^{r}}G_{r}(r_{s},X_{s}^{C})=e^{-\frac{r-r_{s}}{a}}G_{r}(r_{s},X_{s}^{C})e^{-\tilde{b}s-\frac{\tilde{\sigma }}{a}W_{s}}\;. \end{array}$$

The Ito isometry proves the stochastic integral above to be a martingale with zero expectation. Thus, taking the expectations on the both sides of the above inequality gives

(12) $$\begin{array}{c} E\left[e^{-U_{t}^{r}}v(r_{t},X_{t}^{C})+\int_{0}^{t}e^{-U_{s}^{r}}\;dC_{s}\right]\leq v\left(r,x\right)\;. \end{array}$$

Consider now the first term in the expectation above. Since *v* is increasing in *x*, one obtains

$$\begin{array}{c} E[e^{-U_{t}^{r}}v(r_{t},X_{t}^{C})]\leq E[e^{-U_{t}^{r}}v\left(r_{t},x+\mu t\right)]\;. \end{array}$$

From Section 3.1, we know that under the measure *Q*, it holds if *r* < r*

$$\begin{array}{ccc} \psi_{1}\left(r\right) & = & e^{-\frac{r-r^{*}}{a}}E_{Q}\left[e^{-b\tau }\right]\leq e^{-\frac{r-r^{*}}{a}},\\ \psi_{2}\left(r\right) & = & E[\int_{0}^{\tau }e^{-U_{s}^{r}}\psi_{1}\left(r_{s}\right)\;ds]\leq \int_{0}^{\infty }e^{-bs}E_{Q}\left[e^{-\frac{r-r_{s}}{a}}e^{-\frac{r_{s}-r^{*}}{a}}\right]\;ds\leq \frac{1}{b}e^{-\frac{r-r^{*}}{a}}; \end{array}$$

analogously for *r* ⩾ r* one obtains ϕ_1_(*r*) ⩽ 1 and $\varphi_{2}\left(r\right)\leq \frac{1}{b}$. Therefore, we can estimate

$$\begin{array}{ccc} v(r_{t},x+\mu t) & = & 1\;{\text{I}}_{\left[r_{t}\geq r^{*}\right]}F\left(r_{t},x+\mu t\right)+1\;{\text{I}}_{\left[r_{t}<r^{*}\right]}G\left(r_{t},x+\mu t\right)\\ & \leq & (1+e^{-\frac{r_{t}-r^{*}}{a}})\left\{x+\mu t+\frac{\mu }{b}+\Delta \right\}\;. \end{array}$$

Using the measure *Q* defined in Section 3.1 and the fact that *r_t_* under *Q* is normally distributed with mean *re* ^− *at*^ + *b*(1 − *e* ^− *at*^) and variance $\frac{{\tilde{\sigma }}^{2}}{2a}\left(1-e^{-2at}\right)$, we obtain the following estimation

$$\begin{array}{ccc} E[e^{-U_{t}^{r}}v(r_{t},X_{t}^{C})] & \leq & E[e^{-U_{t}^{r}}(1+e^{-\frac{r_{t}-r^{*}}{a}})]\left\{x+\mu t+\frac{\mu }{b}+\Delta \right\}\\ & \leq & e^{-\frac{r}{a}-bt}E_{Q}[(e^{\frac{r_{t}}{a}}+e^{\frac{r^{*}}{a}})]\left\{x+\mu t+\frac{\mu }{b}+\Delta \right\}\\ & \leq & e^{-bt}e^{-\frac{r-b}{a}\left(1-e^{-at}\right)+\frac{{\tilde{\sigma }}^{2}}{4a^{3}}\left(1-e^{-2at}\right)}\left\{x+\mu t+\frac{\mu }{b}+\Delta \right\}\;. \end{array}$$

Also, it holds

$$\begin{array}{c} E\left[\int_{0}^{t}e^{-U_{s}^{r}}\;dC_{s}\right]\leq \int_{0}^{\infty }E[e^{-U_{s}^{r}}]\;dX_{s}<\infty \;, \end{array}$$

so that we can let *t* → ∞ in (12), and obtain by Lebesgue's dominated convergence theorem

$$v\left(r,x\right)\geq E\left[\int_{0}^{\infty }e^{-U_{s}^{r}}\;dC_{s}\right]\;.$$

Obviously, the strategy C* is an admissible one.

**Figure 2.**
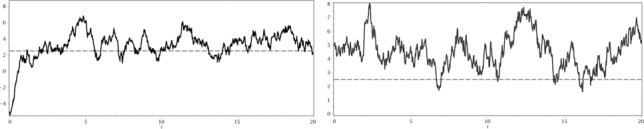
Realizations of an OU-process with starting values *r*_0_ = −5 (the left picture) and *r*_0_ = 5 and the optimal barrier r* = 2.4936 (dashed line).

**Figure 3.**
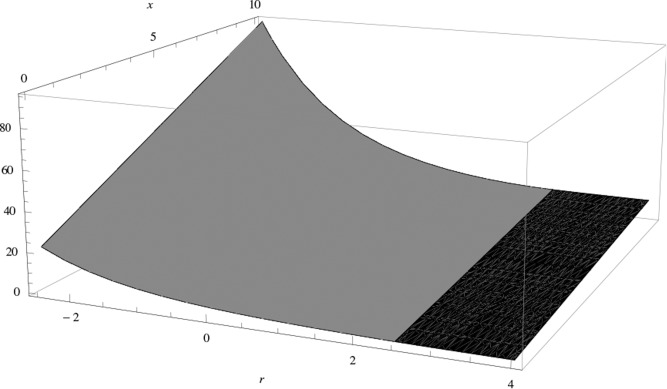
The value function *V*(*r*, *x*). The black and gray areas describe the strategies “maximal consumption” and “no consumption” correspondingly.

Example 3.1. Let *a* = 1, $\tilde{\sigma }=2$ and $\tilde{b}=4$. The function $H\left(r\right)=\frac{{\tilde{D}}_{b/a+1}\left(\frac{b-r}{\sigma }\right)}{{\tilde{D}}_{b/a}\left(\frac{b-r}{\sigma }\right)}$ is strictly increasing and attains $\frac{\sigma }{b}=\frac{1}{2\sqrt{2}}$ at r* = 2.4936. In the time intervals where the process {*r_t_*} attains values smaller than r* it is optimal to wait, in the intervals where *r_t_* ⩾ r* we pay everything. In Figure 2, one can see realizations of an Ornstein–Uhlenbeck process (OU-process) with starting values *r*_0_ = 5 and *r*_0_ = −5. Using the results from Section 3.1 and solving differential equations (4) and (5) with corresponding boundary conditions, we can calculate the value function *V*(*r*, *x*), illustrated in Figure 3.
